# Ca^2+^ Cycling in Heart Cells from Ground Squirrels: Adaptive Strategies for Intracellular Ca^2+^ Homeostasis

**DOI:** 10.1371/journal.pone.0024787

**Published:** 2011-09-14

**Authors:** Xiao-Chen Li, Ling Wei, Guang-Qin Zhang, Zai-Ling Bai, Ying-Ying Hu, Peng Zhou, Shu-Hua Bai, Zhen Chai, Edward G. Lakatta, Xue-Mei Hao, Shi-Qiang Wang

**Affiliations:** 1 State Key Lab of Biomembrane and Membrane Biotechnology, College of Life Sciences, Peking University, Beijing, China; 2 Research Division of Pharmacology, China Pharmaceutical University, Nanjing, China; 3 Laboratory of Cardiovascular Science, National Institute on Aging, Baltimore, Maryland, United States of America; University of Queensland, Australia

## Abstract

Heart tissues from hibernating mammals, such as ground squirrels, are able to endure hypothermia, hypoxia and other extreme insulting factors that are fatal for human and nonhibernating mammals. This study was designed to understand adaptive mechanisms involved in intracellular Ca^2+^ homeostasis in cardiomyocytes from the mammalian hibernator, ground squirrel, compared to rat. Electrophysiological and confocal imaging experiments showed that the voltage-dependence of L-type Ca^2+^ current (*I*_Ca_) was shifted to higher potentials in ventricular myocytes from ground squirrels vs. rats. The elevated threshold of *I*_Ca_ did not compromise the Ca^2+^-induced Ca^2+^ release, because a higher depolarization rate and a longer duration of action potential compensated the voltage shift of *I*_Ca_. Both the caffeine-sensitive and caffeine-resistant components of cytosolic Ca^2+^ removal were more rapid in ground squirrels. Ca^2+^ sparks in ground squirrels exhibited larger amplitude/size and much lower frequency than in rats. Due to the high *I*_Ca_ threshold, low SR Ca^2+^ leak and rapid cytosolic Ca^2+^ clearance, heart cells from ground squirrels exhibited better capability in maintaining intracellular Ca^2+^ homeostasis than those from rats and other nonhibernating mammals. These findings not only reveal adaptive mechanisms of hibernation, but also provide novel strategies against Ca^2+^ overload-related heart diseases.

## Introduction

Intracellular Ca^2+^ regulates a wide variety of cellular processes, including excitation-contraction (E-C) coupling, gene expression, enzyme regulation, cell growth and cell death [1]–[3]. The intracellular Ca^2+^ concentration ([Ca^2+^]_i_) needs be regulated precisely to ensure homeostatic operation of physiological systems. Impaired regulation of intracellular Ca^2+^ under pathologic, ischemic and hypothermic conditions leads to cellular dysfunction and global disorders. For example, the inability to elevate [Ca^2+^]_i_ to a required level following an excitation is a major pathogenic mechanism involved in heart failure [1], [4]. On the other hand, excessive elevation of intracellular Ca^2+^, a status known as Ca^2+^ overload, has been proven to be deleterious to almost all cell types, and can be associated with either necrotic or apoptotic cell death [2], [3], [5]. In the heart, abnormal handling of intracellular Ca^2+^ may induce severe arrhythmias and ventricular fibrillation [6]–[8].

Heart tissues from hibernating mammals demonstrate enhanced resistance against many insulting factors that cause heart dysfunction in humans and non-hibernators [9], [10]. Hibernating mammals, which usually maintain their body temperature around 37°C, can down-regulate their body temperature to only a few degrees in winter [11]. In contrast to the heart arrest and severe arrhythmia at low body temperatures in humans and non-hibernating mammals [9], [12], [13], circulation and respiration are well maintained, although at much lowered rates, in hibernating mammals during hibernation [11], [12]. Moreover, hibernators also exhibit functional stability against pathologic or stressful stimuli in general. For example, in hedgehog hearts, epicardial application of aconitine, administration of high concentration of CaCl_2_, combined procaine and adrenaline treatment or ligation of coronary artery branches cannot trigger the ventricular fibrillation that would commonly occur in non-hibernator hearts [9]. Ground squirrels can survive the 4.5% low oxygen that is fatal for rats [14]. It is therefore suggested that the cardiovascular system of hibernating mammals is a nature's model for arrhythmia resistance and hypoxia tolerance [9], [10]. Therefore, Understanding the adaptive mechanisms of hibernation is not only of important biological significance, but will also provide strategies for solving medical problems.

Given these unique adaptations, it is intriguing how the intracellular Ca^2+^ homeostasis is maintained against so many pathogenic/insulting factors. In heart cells, most of the Ca^2+^ cycling components are involved in E-C coupling, where the Ca^2+^ influx thorough the voltage gated L-type Ca^2+^ channels (*I*_Ca_) activates the Ca^2+^ release from the ryanodine receptors (RyRs) on the sarcoplasmic reticulum (SR) [1], [15]. Ca^2+^ entering the cytosol is either taken back to the SR by the sarcoplasmic/endoplasmic reticulum Ca^2+^-ATPase (SERCA), or removed via Na^+^/Ca^2+^ exchange and other Ca^2+^ transporting systems [16], [17]. A few studies have shown that hibernators tend to down-regulate the L-type Ca^2+^ current and up-regulate the SR Ca^2+^ uptake and release capacity when they hibernate [18]–[21]. These findings demonstrat that a coordinated remodeling of cellular Ca^2+^ handling is a part of the adaptive preparation for maintaining forceful contraction under hibernating conditions. However, previous studies have not fully explained why hibernating mammals, even in their awake state, are still resistant against arrhythmogenic and hypoxic perturbations.

In order to test the hypothesis that hibernators are adapted for better maintaining intracellular Ca^2+^ homeostasis than other mammals, we systemically characterized the Ca^2+^ cycling and E-C coupling processes in heart cells from ground squirrels in comparison with commonly used animal models.

## Results

### Intracellular Ca^2+^ homeostasis under varying temperature

**Figure 1:**
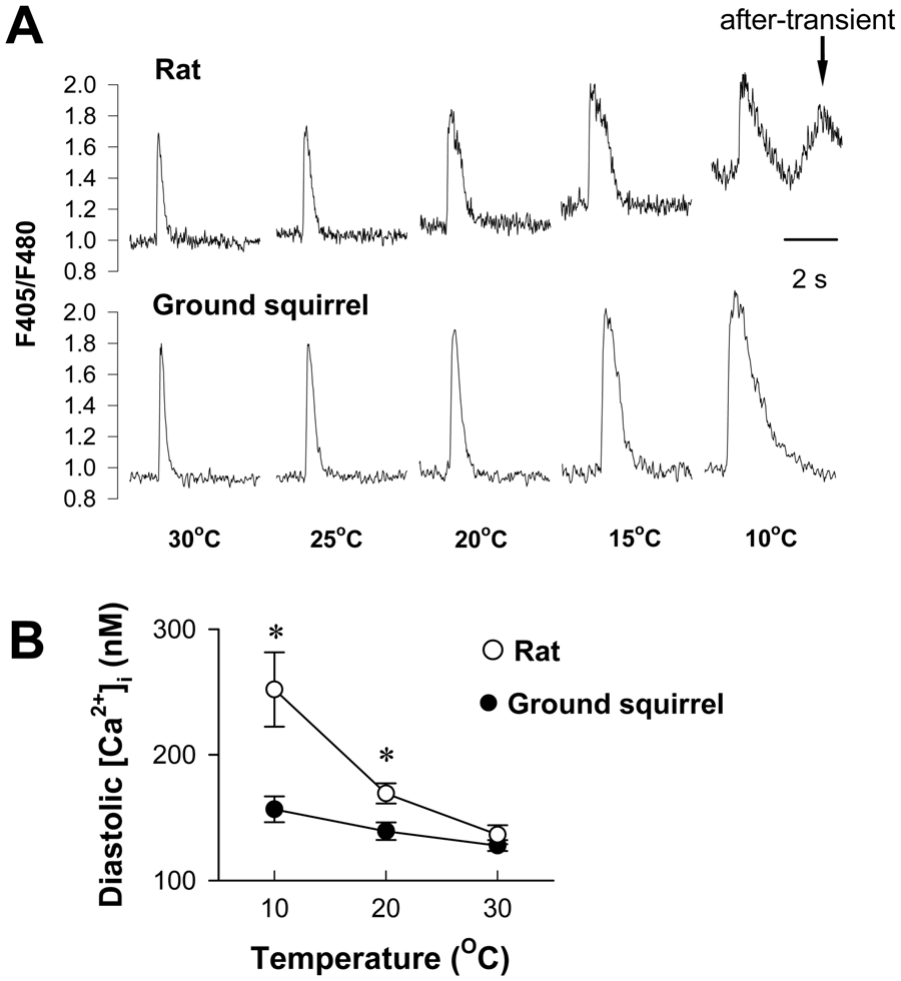
Temperature-dependence of Ca^2+^ transient in ventricular myocytes from rats and ground squirrels. (A) Typical calcium transients presented as the ratio of fluorescence at 405 nm vs. 480 nm (F405/F480). The arrow denotes an “after-transient”. (B) Diastolic [Ca^2+^]_i_ of the Ca^2+^ transients in rat cells (n = 9 cells from 5 animals) were significantly higher than that in ground squirrel cells (n = 5 cells from 3 animals) at low temperatures. *P<0.05.

The intracellular Ca^2+^ dynamics during field stimulation in ventricular myocytes from rats and ground squirrels at varying temperature are illustrated in Figure 1A. The Ca^2+^ transients exhibited an increased background and decreased amplitude in rat cells but not in ground squirrel cells when the temperature was lowered from 30°C to 10°C. Below 15°C, most Ca^2+^ transients were accompanied by “after-transients” (Figure 1A arrow) in rats, which were never observed in ground squirrels. Calibration of indo-1 fluorescence showed that the diastolic Ca^2+^ level was increased from 136±7 nmol/L at 30°C to 252±29 nmol/L at 10°C in rats but was changed little in ground squirrels (Figure 1B). As a consequence, myocardium from ground squirrels was able to maintain vigorous contractile strength with complete relaxation at the low temperature, and avoided the low temperature-induced incomplete relaxation and “after-contractions” that occurred in rat myocardium (Figure S1). These results provided direct evidence that beating heart cells from hibernating mammals are able to keep homeostatic intracellular Ca^2+^ at varying temperatures.

### Ca^2+^ influx and membrane potential

**Figure 2:**
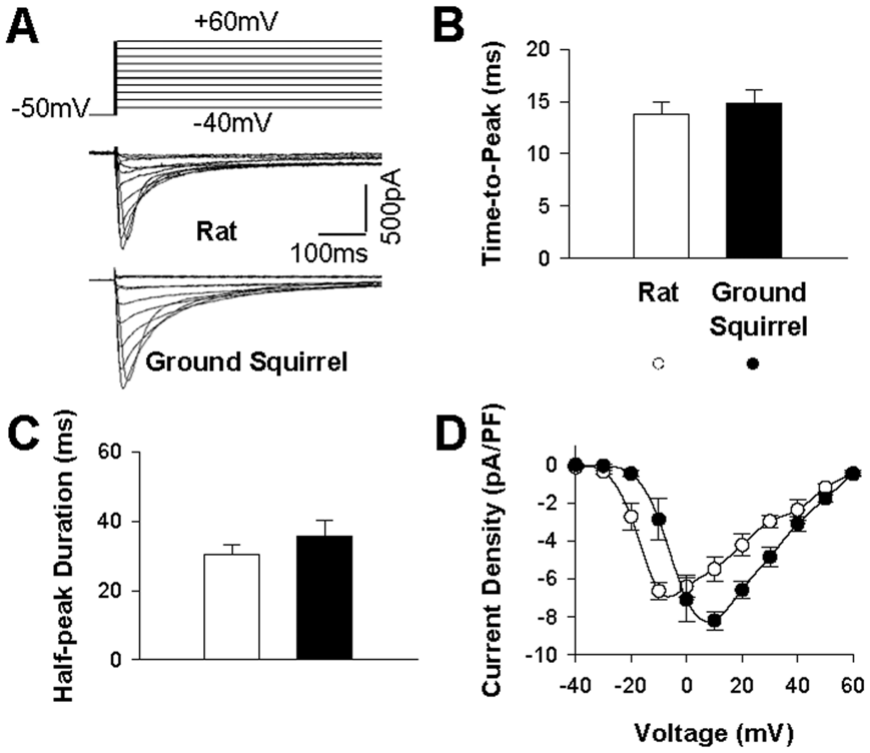
Voltage-dependence of Ca^2+^ current. (A) Ca^2+^ currents were activated by depolarizations from a holding potential of −50 mV to test potentials ranging from −40 mV to +60 mV with 10-mV steps. (B) The time-to-peak and (C) Half-peak duration was measured at 0 mV. (D) The current-voltage (*I*_Ca_−*V*_m_) relation in rats (n = 5 from 5 animals) and ground squirrels (n = 5 from 4 animals).

In cardiac cells, *I*_Ca_ through L-type Ca^2+^ channels is the major pathway of Ca^2+^ entry, and is also the trigger for Ca^2+^-induced Ca^2+^ release from the sarcoplasmic reticulum. We used the whole-cell patch clamp technique to record *I*_Ca_ in single ventricular myocytes when the membrane was depolarized to various voltages (*V*_m_) from the holding potential of −50 mV (Figure 2A). We included in the pipette 10 mmol/L EGTA to avoid Ca^2+^-induced feedback to *I*_Ca_. Measurement of the time course of *I*_Ca_ showed that the time-to-peak (Figure 2B) and half-peak duration (Figure 2C) were similar in ground squirrels and rats, indicating similar activation and inactivation kinetics in these two species. The *I*_Ca_−*V*_m_ curves were both bowl-shaped crossing the same reversal potential (Figure 2D). However, the voltage-dependence of *I*_Ca_ differed. Both the activation threshold and the peak activation potential were shifted ∼10 mV more positive in ground squirrels than in rats. As the results, the *I*_Ca_ amplitude at 20 mV was about one order of magnitude lower in ground squirrels than in rats.

**Figure 3:**
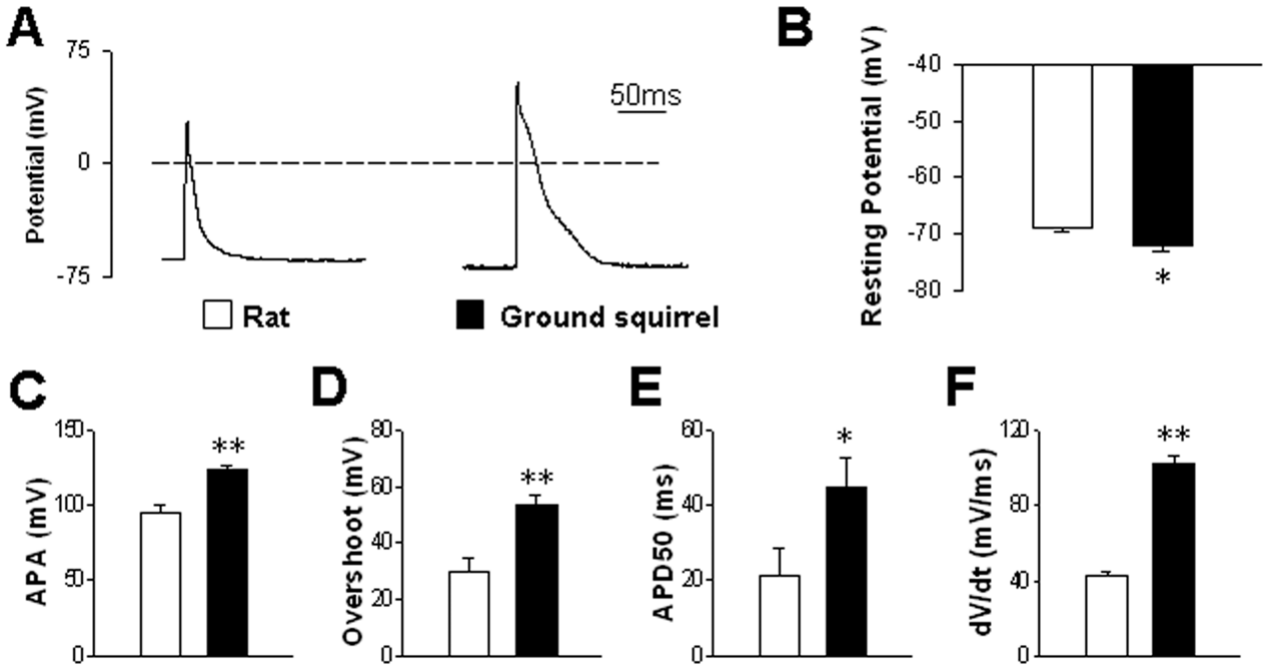
Action potentials evoked by current pulses. (A) Typical examples of action potentials in ventricular myocytes from rats (left) and ground squirrels (right). (B) Resting membrane potentials were compared between rats (n = 18 cells from 7 animals) and ground squirrels (n = 21 cells from 7 animals). (C) Action potential amplitude (APA), (D) overshoot, (E) half-repolarization duration (APD50) and (F) maximum dV/dt of depolarization were compared between rats (n = 7 cells from 5 animals) and ground squirrels (n = 6 cells from 5 animals). *P<0.05 and **P<0.01.

The right-shift of *I*_Ca_−*V*_m_ curve in ground squirrel cells tends to result in later activation of *I*_Ca_ during an action potential. To examine whether the ground squirrel action potential is indeed coordinated with the voltage dependence of *I*_Ca_, we performed whole-cell current clamp experiments (Figure 3A). When the pipette current was clamped at zero, we recorded resting potentials around −70 mV in both cell types (Figure 3B).

Upon 2-ms current pulse stimulation, action potentials firing in ground squirrel cells shared similar triangle morphology as in rat cells (Figure 3A) and most other rodent cardiomyocytes [22]. Notably, the amplitude of action potential (APA) was significantly higher in ground squirrels (Figure 3C). The higher APA was basically attributable to their much larger overshoot (Figure 3D). Also, ground squirrel cells exhibited longer half-repolarization duration (Figure 3E) and faster depolarization rate (dV/dt) (Figure 3F). Larger depolarization rate and longer duration both facilitate more robust activation of the high-threshold *I*_Ca_ in ground squirrels.

### Evoked and spontaneous Ca^2+^ release

**Figure 4:**
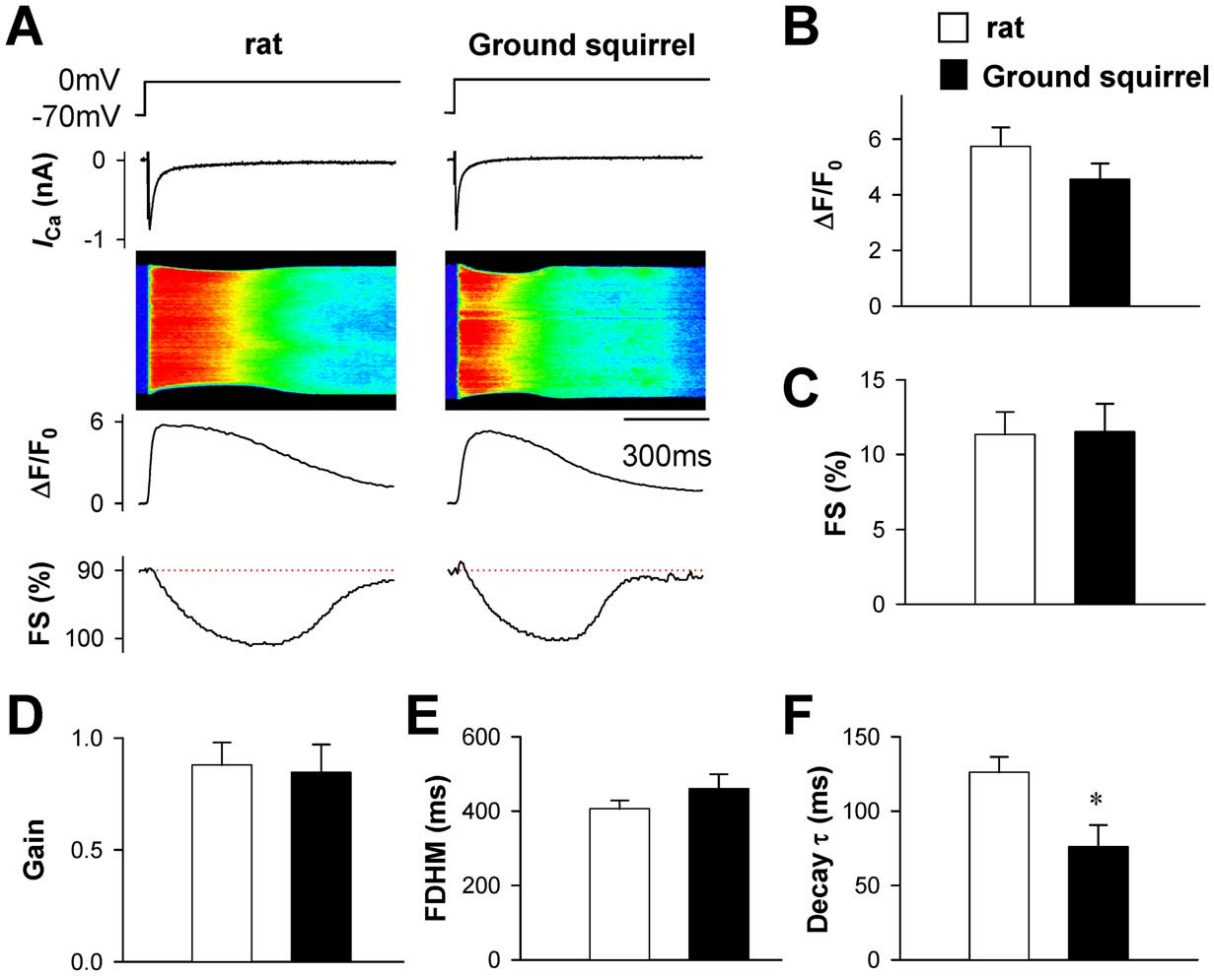
Ca^2+^ transients evoked by depolarization to 0 mV. (A) Typical examples of simultaneous recording of *I*_Ca_ and Ca^2+^ transient (image and ΔF/F_0_) in ventricular myocytes from rats (left) and ground squirrels (right). The fractional shortening (FS) was determined by edge-detection of Ca^2+^ transients. (B) The amplitude of Ca^2+^ transient (ΔF/F_0_) and (C) the fractional shortening (FS) reflecting the amplitude of contraction were compared between rats (n = 12 cells from 8 animals) and ground squirrels (n = 16 cells from 8 animals). (D) The gain of E-C coupling was calculated as the ratio between ΔF/F_0_ and *I*_Ca_ density. (E) The full-duration at half-maximal (FDHM) and (F) the time constant (τ) of second half decay of Ca^2+^ transient were compared between rats (n = 12 cells from 8 animals) and ground squirrels (n = 16 cells from 8 animals).

To compare the *I*_Ca_-evoked Ca^2+^ release from the sarcoplasmic reticulum, we recorded the Ca^2+^ transients by confocal line-scan imaging (Figure 4A) when the cells were depolarized to 0 mV. Given that the *I*_Ca_ density was similar between two species (data not shown, but similar to Figure 2C), the amplitude of Ca^2+^ transients (Figure 4B) and fractional cell shortening (Figure 4C) were basically comparable between rats and ground squirrels. The gain of E-C coupling, defined as the Ca^2+^ transient amplitude per unit *I*_Ca_ density, was also about the same (Figure 4D). Notably, although the full-duration at half-maximum (FDHM) was similar between two species (Figure 4E), the time constant of the second half of the Ca^2+^ transient decay was significantly shorter in ground squirrels than in rats (Figure 4F).

**Figure 5:**
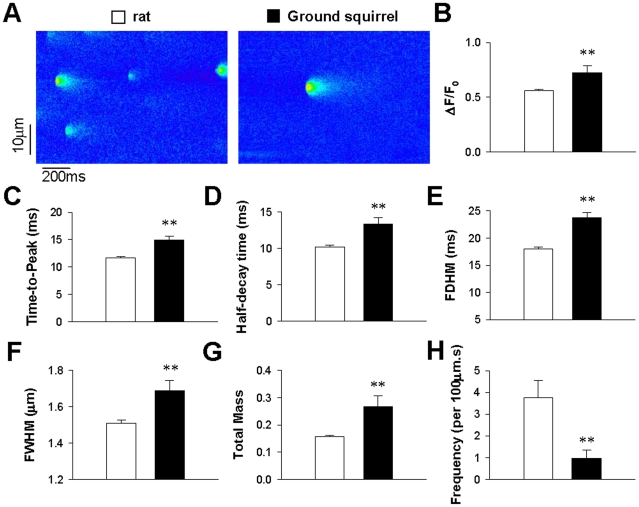
Spontaneous Ca^2+^ sparks. (A) Line-scan images of spontaneous Ca^2+^ sparks in ventricular myocytes from rats (left) and ground squirrels (right). (B) The spark amplitude (ΔF/F_0_). (C) The time-to-peak. (D) The half-decay time. (E) The full duration at half-maximum (FDHM). (F) The full width at half-maximum (FWHM). (G) The total signal mass presented in arbitrary units (A.U.). (H) The frequency of Ca^2+^ sparks calculated by dividing the total number of sparks by the product of distance (µm) and time (s) of line scanning. **P<0.01 (n = 270 sparks in 6 rats and 53 sparks in 5 ground squirrels).

Although the global Ca^2+^ release following an *I*_Ca_ trigger appeared similar in two species, the characteristics of local RyR Ca^2+^ release, as reflected in Ca^2+^ sparks [23], was diffeent. Confocal imaging of quiescent ventricular myocytes (Figure 5A) showed that spontaneous Ca^2+^ sparks in ground squirrels exhibited higher amplitude than in rats (Figure 5B). The higher amplitude was at least partially attributable to prolonged RyR Ca^2+^ release, as reflected by the longer time-to-peak (Figure 5C). As the half-decay time of Ca^2+^ spark was also prolonged (Figure 5D), the FDHM in ground squirrels was much longer than that in rats (Figure 5E). Prior to the present study, Ca^2+^ sparks in rats had been shown to be brighter than other animal species. Here, Ca^2+^ sparks in ground squirrels exhibited an even larger full-width at half-maximum (FWHM, Figure 5F) and signal mass (Figure 5G), and thus may represent the largest Ca^2+^ spark among all characterized mammal ventricular cells. The larger spark size in ground squirrels was partially due to higher SR Ca^2+^ load than in rats (Figure S2).

Although spontaneous Ca^2+^ sparks in ground squirrels are brighter, the Ca^2+^ spark frequency was less than 1/4 that in rats (Figure 5H). The lower frequency of Ca^2+^ sparks reflects less frequent leak from the SR Ca^2+^ store, which is essential for intracellular Ca^2+^ homeostasis.

### Ca^2+^ removal

**Figure 6:**
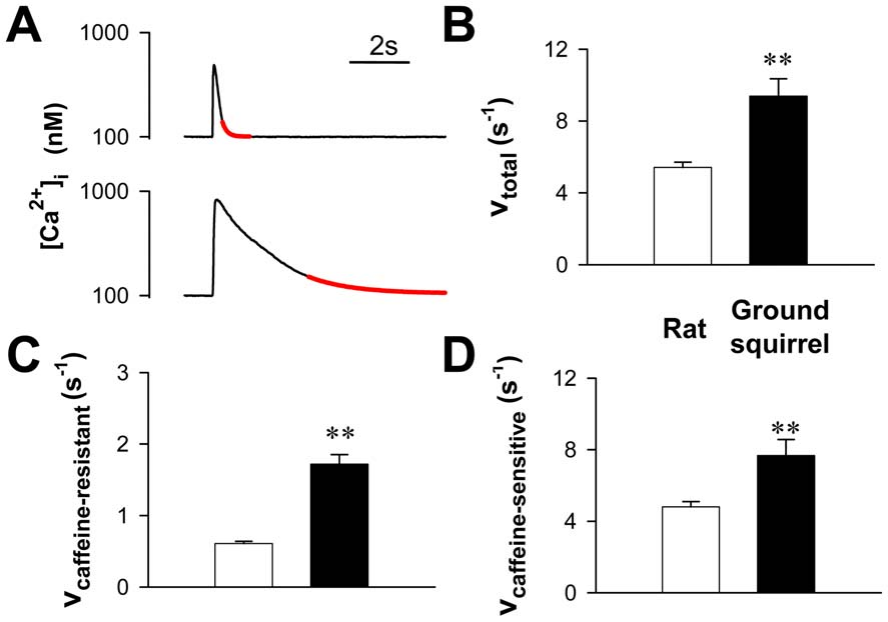
Analysis of Ca^2+^ removal mechanisms. (A) Representative Ca^2+^ transients induced by electrical stimulation (upper) and 20 mmol/L caffeine (lower) in a rat cardiac myocyte. The red lines illustrate the fitting of the lower 20% of Ca^2+^ transients. (B) Rate constants of total Ca^2+^ removal. (C) Rate constants of the caffeine-resistant component. (D) Rate constants of the caffeine-sensitive component were calculated as the difference between the total and the caffeine-resistant component. ** *P*<0.01 (n = 19 cells from 3 rats and 14 cells from 3 ground squirrels).

During the contraction-relaxation cycle, timely removal of Ca^2+^ from cytosol is important for intracellular Ca^2+^ homeostasis. During the whole-cell voltage clamp, the faster decay of Ca^2+^ transients in ground squirrel cells suggested a more rapid clearance of intracellular Ca^2+^ than in rat cells (Figure 4F). Under field stimulation conditions, the late decay of Ca^2+^ transients (Figure 6A upper) also exhibited a higher rate constant in ground squirrels than in rats (Figure 6B). To assess the mixed contributions of SERCA pumping, Na^+^/Ca^2+^ exchange, mitochondrial Ca^2+^ uptake and other possible mechanisms [16], we blocked SR Ca^2+^ uptake by perfusing the cells with 20 mmol/L caffeine (Figure 6A lower). The caffeine-resistant component of Ca^2+^ removal comprised a minor part in the total Ca^2+^ removal and was similar in both species (Figure 6C). Thus, the major species difference of Ca^2+^ removal was due to the higher rate of caffeine-sensitive SR Ca^2+^ uptake (Figure 6D), which reflected higher SERCA activity in ground squirrel cells.

### Relationship between intracellular Ca^2+^ and contraction

**Figure 7:**
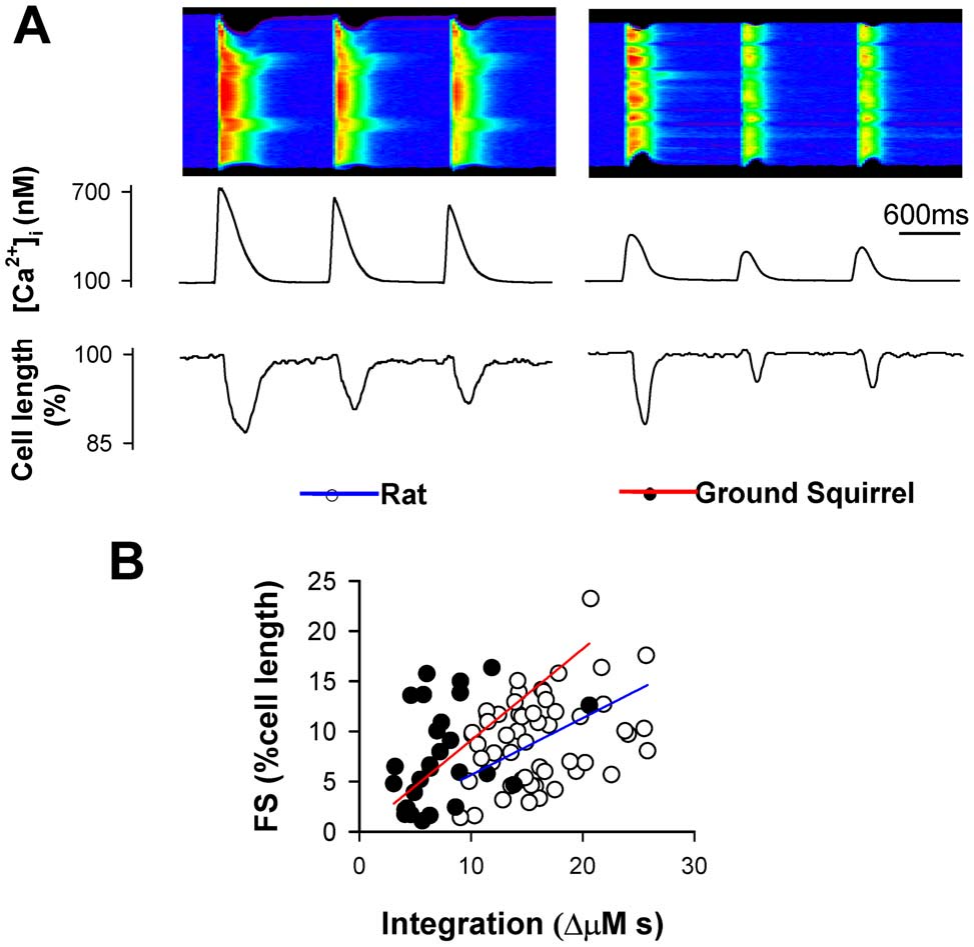
Relationship between Ca^2+^ transients and cell shortening. (A) Ca^2+^ transients (upper image and plot) and cell length (lower plot) in response to 1 Hz electrical field stimulation in rat (left) and ground squirrel (right) cells. (B) The fractional shortening (FS) of each contraction was plotted against the integration of Ca^2+^ transients. Color lines indicate linear regression.

In cardiomyocytes from both species, repetitive field stimulation at 1-s intervals evoked a series of Ca^2+^ transients/contractions with varying amplitude (Figure 7A). To examine whether the myofiliments in ground squirrels are more sensitive to Ca^2+^ in generating a contraction, we investigated the relationship between cell shortening and Ca^2+^ transient integral (Figure 7B). Linear regression indeed revealed a higher contraction/transient ratio in ground squirrels, suggesting a higher Ca^2+^ signaling efficiency in generating contractions.

## Discussion

### Strategies of adaptation

Hibernation involves multiple insulting factors that are fatal for non-hibernating mammals, such as violent change of body temperature, sustained deep hyperthermia during hibernation, as well as intensified sympathetic innervation during arousal. It is therefore intriguing to understand why the cardiovascular system of hibernating mammals can maintain functional stability against these extreme factors. In the present study, we have for the first time systemically compared major Ca^2+^ cycling processes and intracellular Ca^2+^ homeostasis between hibernating and nonhibernating species. Compared to those in rats and most other mammals, heart cells from ground squirrels have evolved several adaptive strategies for more optimal and efficient maintenance of intracellular Ca^2+^ homeostasis:

#### 1) Elevated threshold for Ca^2+^ entry

Excessive Ca^2+^ entry is a major reason of intracellular overload in many pathogenic processes [2], [3], [5]. In the deep hyperthermia condition during hibernation, the resting potential declines due to slowed turnover rate of sodium pumps and compromised transmembrane ionic gradients [24]. Therefore, our finding on the right-shift of *I*_Ca_−*V*_m_ curve in ground squirrels has significant adaptive significance. The high threshold of *I*_Ca_ would lower the risk of spontaneous *I*_Ca_ activation, and is thus helpful in preventing excessive Ca^2+^ entry during the resting state. As E-C coupling highly depends on the profile of action potentials [25], the higher threshold of *I*_Ca_ would be expected to compromise the activation of E-C coupling. Interestingly, there seems to have been a co-evolution between L-type Ca^2+^ channels and action potentials in ground squirrels. The more negative diastolic potential enhances L-type channel availability; the higher depolarization rate accelerates channel activation; and the longer duration of action potential increases Ca^2+^ influx. These factors well compensates the right-shift of *I*_Ca_−*V*_m_ curve, and adaptively corrects the probability of Ca^2+^ channel activation.

#### 2) Suppressed leak from Ca^2+^ store

Ca^2+^ leak from intracellular Ca^2+^ stores is another major factor interfering with intracellular Ca^2+^ homeostasis [8], [16]. A higher leakage imposes a heavier burden on energy-based Ca^2+^ transports [26]. In situations of compromised metabolism, such as hypothermia or recovery from hypoxia, excessive Ca^2+^ leak triggers a vicious cycle of Ca^2+^-induced Ca^2+^ release, and leads to irreversible process of Ca^2+^ mishandling and cell damage [26]. In heart cells from ground squirrels, spontaneous spark frequency is quite low. Even taking into consideration the larger Ca^2+^ leak signal mass in ground squirrels, the ensemble Ca^2+^ spark signal mass per unit time, i.e. the product of individual spark signal masse and spark frequency, is only about 1/3 of that in rat cells. The suppressed SR Ca^2+^ leak in ground squirrel cells is an important part of the Ca^2+^ homeostatic adaptation.

#### 3) Accelerated rate of cytosolic Ca^2+^ removal

In essence, intracellular Ca^2+^ homeostasis is a balance between continuous Ca^2+^ removal from the cytosol and continuous Ca^2+^ entry/leak. In addition to the higher threshold of Ca^2+^ entry from L-type Ca^2+^ channels and less RyR Ca^2+^ leak from the SR, cardiomyocytes from ground squirrels also exhibit faster removal rate of cytosol Ca^2+^. During a Ca^2+^ transient, the caffeine-sensitive component of Ca^2+^ uptake by SERCA is no doubt the major driving force behind the faster Ca^2+^ removal in ground squirrels.

In addition to SR Ca^2+^ pumping, the caffeine-resistant component of Ca^2+^ removal, although secondary in the decay of a Ca^2+^ transient, plays an important role in maintaining intracellular Ca^2+^ homeostasis during the resting state [16]. This component usually involves Na^+^/Ca^2+^ exchanger and Ca^2+^-ATPase on the cell membrane, as well as Ca^2+^ uptake by mitochondria [16]. Although it is not known whether ground squirrels have additional Ca^2+^ transport mechanisms, their caffeine-insensitive component appears to be much more powerful than in rats. During hibernation, the heart rate drops to several beats per minute [11]. It is therefore expected that the stronger caffeine-insensitive Ca^2+^ transports are of great adaptive importance in maintaining Ca^2+^ homeostasis between the slow heart beats.

#### 4) Enhanced Ca^2+^ signaling efficiency in generating contraction

For a functional reserve during hibernation, cells of hibernating mammals need to keep diastolic Ca^2+^ sufficiently low and systolic Ca^2+^ sufficiently high. In the context of temperature-dependent decrease of Ca^2+^ transport activities, these two demands become even more challenging. Ground squirrels gain a higher Ca^2+^ signaling efficiency in generating contractile strength by enhanced Ca^2+^ sensitivity of myofiliments. This adaptation not only lowers the Ca^2+^ requirement and its associated energy burden in generating sufficient pumping power, but also partially compensates the temperature-dependent decrease of myofiliment Ca^2+^ sensitivity [27] under hypothermic conditions, and is thus important for surviving hibernation.

### Medical significance

Excessive elevation of resting Ca^2+^ has been proven to be deleterious to almost all cell types, and can be associated with either necrotic or apoptotic cell death [3], [5]. Abnormal handling of intracellular Ca^2+^ in the heart may induce severe arrhythmias and ventricular fibrillation [6]–[8]. The adaptive capability to maintain intracellular Ca^2+^ homeostasis in hibernator cells is beneficial in the following pathophysiological states:

#### Anti-arrhythmia

Many severe arrhythmias are caused by intercellular mishandling of intracellular Ca^2+^ homeostasis [6]–[8]. For example, leaky RyRs due to mutations underlies the catecholaminergic polymorphic ventricular tachycardia [8]. Intracellular Ca^2+^ overload usually leads to after-depolarizations, which is a major mechanism of arrhythmogenisis [16]. In contrast, hearts from the hedgehog, another hibernating mammal, are resistant to arrhythmogenic insults, including application of aconitine, high concentration extracellular calcium, procaine or adrenaline [9]. Our results show that cardiomyocytes from ground squirrels was able to avoid the low temperature-induced “after-transients” that occurs in rat hearts. The anti-arrhythmic property of the hibernator heart appears to be tightly linked to their Ca^2+^ homeostatic mechanisms, as increasing RyR leak and interfering Ca^2+^ uptake by low dose of caffeine can reproduce the arrhythmic after-contractions in ground squirrels [12].

#### Surgery and transplantation

Hypothermic anesthesia is often used in by-pass surgery to lower the demand of blood supply [28]. The American Heart Association suggests that the body temperature during surgery should be controlled above 30°C, because further decrease of body temperature will cause ventricular fibrillation and irreversible damage of organ function [29]. In contrast, hibernating mammals are able to regulate their body temperature between 37°C and freezing point without endangering their life. The ability for hibernator cells to keep intracellular Ca^2+^ homeostasis is one of the key mechanisms underlying their tolerance of hyperthermia. Also, organ preservation under low temperature is a major challenge in transplantation [28]. Intracellular Ca^2+^ homeostasis is again a key issue limiting the time window for organ transplantation [30]. Therefore, hyperthermia-resistant mechanisms in hibernating mammals, including those involved in intracellular Ca^2+^ homeostasis, are expected to provide strategies for improving the clinical practice in hypothermic anesthesia and organ preservation. One successful example is that a hibernation-inducing trigger obtained from hibernating woodchucks prolongs the survival time of auto-perfused canine multiorgan preparation, including working heart, lung, kidney, etc., from an average of 16 hours to 43 hours [31]. This study produced one of the longest average survival times for organ preservation.

In 2007, the entire genome of thirteen-lined ground squirrels (*Spermophilus tridecemlineatus*) was roughly sequenced, and deeper sequencing is ongoing. With the application of modern research tools on hibernation research, the mechanisms underlying the adaptation of hibernating mammals, including those involved in Ca^2+^ homeostasis, will be understood in more detail. These mechanisms will be expected to provide new ideas for developing therapeutic strategies against diseases/disorders involving intracellular Ca^2+^ mishandling.

## Materials and Methods

### Ethics Statement

The investigation conforms with the Guide for Care and Use of Laboratory animals published by the US National Institutes of Health. Animal trapping and experiments were approved by the Institutional Animal Care and Use Committee of Peking University (Permit Numbers: lsc-wangsq-1 and lac-tianyl-2). All surgery was performed under 20% ethylurethanm (i.p. injection 5 ml/kg) anaesthesia, and all efforts were made to minimize suffering.

### Cell preparation

The ground squirrel used in the present study, *Citellus dauricus*, is a species dominant in Northeast Asia, which belongs to the same genus as those live in North America, e.g., *Spermophilus tridecemlineatus*. The animals were trapped in the field of Zhanbei County in Hebei Province, China. Ground squirrels were kept in active state in an environment of natural photoperiod at room temperature. The Sprague-Dawley rats were housed by and purchased from the Laboratory Animal Center of Peking University. The hearts were rapidly excised from adult ground squirrels (200–250 g) or rats (200–250 g) under anaesthesia and mounted on a Langendorff apparatus. Ventricular cardiac myocytes from animals were enzymatically isolated as previously reported [32]. Myocytes were stored in a solution containing (in mM) 137 NaCl, 4.0 KCl, 1.0 CaCl_2_, 1.2 MgCl_2_, 1.2 NaH_2_PO_4_, 10 glucose and 10 HEPES, pH 7.35 adjusted with NaOH, and were used the same day they were isolated. The functional features of the prior non-hibernating state were assumed to be preserved after cell isolation, because isolated cardiomyocytes from hibernating and non-hibernating individuals exhibited distinct functional performances [21] that were in good agreement with those observed in cardiac tissues [18]–[20].

### Measurement of intracellular Ca^2+^ concentration

When indo-1 fluorescence was used to determine intracellular Ca^2+^ concentration, cells were incubated in Tyrode's solution containing 2.5 µmol/L indo-1 AM for about 10 min in the dark at 37°C. The fluorescence was exited by a UV laser and measured by ASCS meridian 575UV confocal microscope using 485 nm and 405 nm band-pass filters as reported previously [33]. Intracellular Ca^2+^ concentration was measured at different temperatures, and calculated with formula [Ca^2+^]_i_ = k_A_(R−R_min_)/(R_max_−R), where k_A_ was an apparent constant determined by calibration [23], R is the ratio of fluorescence at 405 nm vs. 485 nm, R_max_ and R_min_ were the R in Ca^2+^ free and Ca^2+^-saturated buffers, respectively.

### Whole-cell patch clamp

Whole-cell Ca^2+^ current were recorded at room temperature with 4 mmol/L 4-aminopyridine and 0.02 mmol/L tetrodotoxin in the bath solution, as reported reviously [34]. The pipettes were filled with (in mmol/L) CsCl 105, MgCl_2_ 5, Na_2_ATP 5, TEA-Cl 15, EGTA 11, CaCl_2_ 1, and HEPES 10 (pH was adjusted to 7.2 with CsOH). When Ca^2+^ transients were recorded simultaneously, the pipette electrode was filled with (in mmol/L) CsCl 127, NaCl 10, MgCl_2_ 1, MgATP 5, TEA-Cl 15, HEPES 10 and fluo-4 pentapotassium 0.2 (pH 7.2 adjusted with CsOH). Leak current subtraction was performed Offline.

Membrane potential was recorded at room temperature under whole-cell current clamp mode. The pipettes were filled with intracellular solution which contained (in mmol/L) KCl 125, MgCl_2_ 6, Na_2_ATP 5, EGTA 0.2 and HEPES 10 (pH was adjusted to 7.2 with KOH). Action potentials were evoked by 2-ms current pulses.

### Confocal imaging of Intracellular Ca^2+^

Ca^2+^ indicators were loaded into myocytes either via the pipette electrode in patch-clamp experiments [34]or otherwise by incubation in a Tyrode's solution containing 20 µmol/L fluo-4 AM or fluo-5F AM (Molecular Probes) at 37°C for 5 min. The fluorescence was measured with Zeiss LSM-510 or LSM-5Live laser scanning confocal microscope (Carl Zeiss, Oberkochen, Germany). Images were acquired at the room temperature (unless otherwise specified) in the line-scan mode. The Ca^2+^ level was either reported as the fluorescence normalized by its resting level (F/F_0_), or calculated according to the formula [Ca^2+^]_i_ = k_d_·R/(k_d_/C_0_+1−R) [35], where R = F/F_0_, k_d_ was set to 1.1 mol/L for fluo-4 and 2.3 mol/L for fluo-5N. The threshold for Ca^2+^ spark detection was set at 1.25 *F/F_0_*. Cell contraction was measured by edge detection and presented as the fractional shortening of cell length.

### Data analysis

The data are presented as means ± s.e.m. Statistical significance was determined using Student-*t* test unless otherwise specified. A *P* value<0.05 was considered to be statistically significant.

## Supporting Information

Figure S1 Contraction of papillary heart muscles in rats (left) and ground squirrels (right) in response to 0.2 Hz field stimulation at 8°C. Note the after-contractions and elevated resting tension in the rat.
(TIF)

Figure S2 SR Ca^2+^ load of ventricular myocytes at room temperature (RT) and 10°C were measured in rats and ground squirrels by perfusing the cells with 20 mmol/L caffeine after 15 min indo-1 AM loading. SR load was reported as the ratio of fluorescence at 405 nm vs. 485 nm.
(TIF)
