# Supplementary figures and images for: Ca2+ Cycling in Heart Cells from Ground Squirrels: Adaptive Strategies for Intracellular Ca2+ Homeostasis
Source: PLoS One. 2011 Sep 14;6(9):e24787. doi: 10.1371/journal.pone.0024787 (PMC3173481; doi:10.1371/journal.pone.0024787)

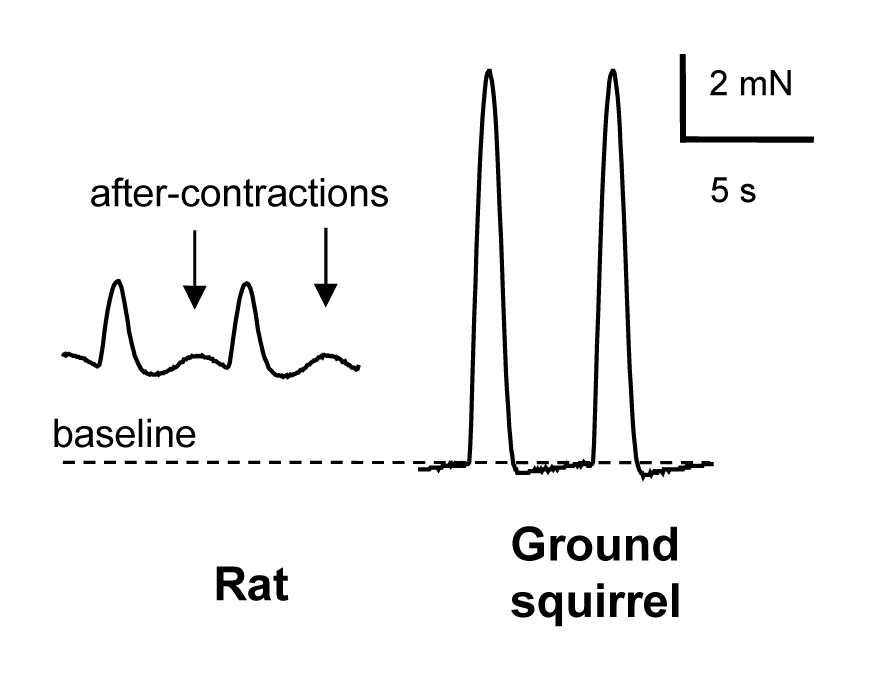

Supplement: Figure S1 — Contraction of papillary heart muscles in rats (left) and ground squirrels (right) in response to 0.2 Hz field stimulation at 8°C. Note the after-contractions and elevated resting tension in the rat. (TIF) [file pone.0024787.s001.tif]

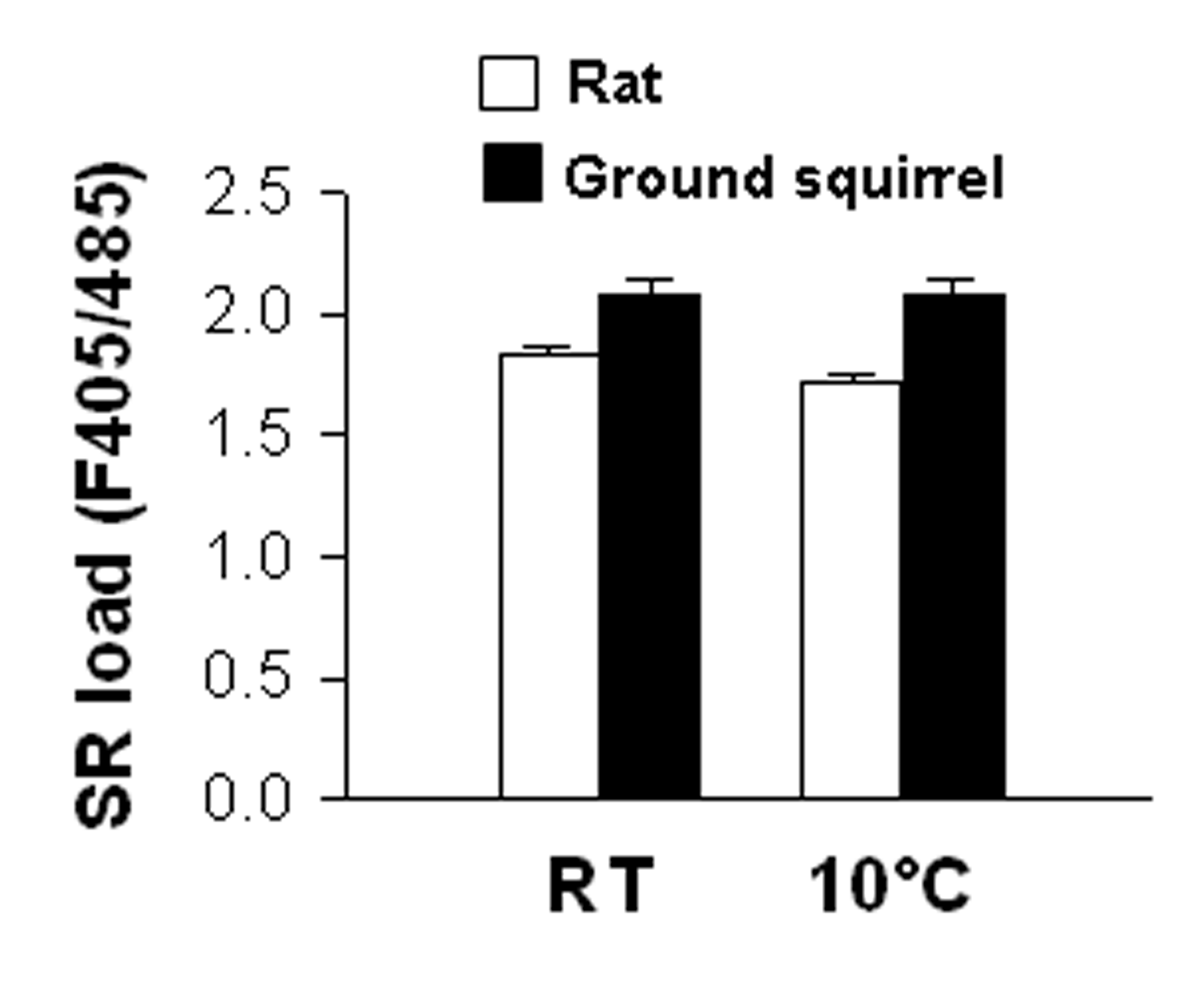

Supplement: Figure S2 — SR Ca2+ load of ventricular myocytes at room temperature (RT) and 10°C were measured in rats and ground squirrels by perfusing the cells with 20 mmol/L caffeine after 15 min indo-1 AM loading. SR load was reported as the ratio of fluorescence at 405 nm vs. 485 nm. (TIF) [file pone.0024787.s002.tif]
